# Age, gender and disability predict future disability in older people: the Rotterdam Study

**DOI:** 10.1186/1471-2318-11-22

**Published:** 2011-05-10

**Authors:** Ümit Taş, Ewout W Steyerberg, Sita MA Bierma-Zeinstra, Albert Hofman, Bart W Koes, Arianne P Verhagen

**Affiliations:** 1 Department of General Practice, Erasmus MC - University Medical Centre Rotterdam, P.O. Box 2040; 3000 CA Rotterdam, the Netherlands; 2 Department of Public Health, Erasmus MC - University Medical Centre Rotterdam, P.O. Box 2040; 3000 CA Rotterdam, the Netherlands; 3 Department of Epidemiology and Biostatistics, Erasmus MC - University Medical Centre Rotterdam, P.O. Box 2040; 3000 CA Rotterdam, the Netherlands

**Keywords:** disability, older people, prediction model, prospective cohort

## Abstract

**Background:**

To develop a prediction model that predicts disability in community-dwelling older people. Insight in the predictors of disability is needed to target preventive strategies for people at increased risk.

**Methods:**

Data were obtained from the Rotterdam Study, including subjects of 55 years and over. Subjects who had complete data for sociodemographic factors, life style variables, health conditions, disability status at baseline and complete data for disability at follow-up were included in the analysis. Disability was expressed as a Disability Index (DI) measured with the Health Assessment Questionnaire.

We used a multivariable polytomous logistic regression to derive a basic prediction model and an extended prediction model. Finally we developed readily applicable score charts for the calculation of outcome probabilities.

**Results:**

Of the 5027 subjects included, 49% had no disability, 18% had mild disability, 16% had severe disability and 18% had deceased at follow-up after six years. The strongest predictors were age and prior disability. The contribution of other predictors was relatively small. The discriminative ability of the basic model was high; the extended model did not enhance predictive ability.

**Conclusion:**

As prior disability status predicts future disability status, interventive strategies should be aimed at preventing disability in the first place.

## Background

Disability, especially in older people, is a common problem and in most cases a chronic condition. Prevalence rates range from 30% for people aged 75 or older to 40% for those aged 85 and older [1].

Older people with disability may become dependent on assistive devices or other people. This may have a negative impact on the quality of their lives. At the end, the level of disability will determine whether older people will be able to live in their own house, with or without modifications, or whether they have to live in a home for older people or nursing home. For the future the expected increase in disability produces economical and logistical challenges for society. There will be a an increasing demand of professional caregivers as most children of older people will not be in the position, either by choice or (economical) necessity, to take care of their own parent(s).

For targeting preventive, curative or palliative strategies it is important that disability can be predicted in order to identify high-risk groups. Prediction of high-risk groups is only helpful when effective preventive strategies can be provided to these groups. Several systematic reviews report on treatment strategies with favourable outcome on disability like the use of memantine in dementia, centre based physical activity programs for older adults with cardiovascular disease, chronic obstructive airway disease or osteoarthritis, strength training in the general older population and vitamin D supplementation to reduce hip fractures [2-5]. There are several other studies on possible preventive strategies like resistance or endurance training, preventive home visits or multidimensional geriatric assessment [6-10].

Nevertheless, there is a clear need for predicting disability. This may also help the individual, the care giving relatives and the related institutions to anticipate to future dependency and improve their policy. There are however, as yet no (comprehensive) prediction models for disability in a general population of older people, including risk factors as well as prognostic factors. Though many individual prognostic factors for worsening disability have already been identified, studies on prognostic models are sparse [11]. Also baseline disability is not always taken into account. Therefore we developed a prediction rule for long-term disability in older people based on a number of easily obtainable predictors including baseline disability status.

## Methods

### Study population and setting

The Rotterdam Study is a population-based prospective cohort study of the incidence and determinants of chronic diseases and disability in older people [12,13]. The Medical Ethics Committee of the Erasmus Medical Centre approved the study. All 10,275 inhabitants of a suburb of Rotterdam aged 55 years or older were invited to participate. A total of 7,983 (78%) men and women consented and entered the study.

At baseline, between July 1989 and June 1993, comprehensive interviews were conducted during home visits by trained researchers followed by further assessments of the participants at the research centre. The interview and assessments were repeated at the first follow-up between 1994 and 1996 and at the second follow-up between 1997 and 1999. The interviews comprised questions on demographic factors, socio-economic status, activities of daily living, cardiovascular diseases, joint complaints, medical history, medical consumption, life events, smoking, medication and family history. The assessments at the research centre included anthropometrical, ophthalmological and biochemical factors.

The present study is carried out with data of the baseline and the second follow-up of the Rotterdam Study, as activities of daily living were not assessed at the first follow-up. The follow-up period therefore comprises about six years.

### Outcome: disability status

For the assessment of disability the Stanford Health Assessment Questionnaire (HAQ) was used [14]. The Health Assessment Questionnaire has proven to be reliable, valid and sensitive to change in both general populations and populations with a specific disease [15]. The HAQ measures disability in eight fields (dressing and grooming, rising, reach, hygiene, eating, walking, grip and activity). Each field comprises two to four items. Per item the status of the respondent is scored as able to do without difficulty (0), with some (1) or much (2) difficulty or unable to do with or without assistance (3). The highest item score determines the final field score. The mean score of all fields constitutes the Disability Index (DI) ranging from 0.0 to 3.0. A person, for example, who experiences only some difficulty in dressing and rising without needing assistance, would be given 2 points. Dividing this by the number of fields with complete information, usually all eight, would give a DI of 0.25. If someone would be unable to perform one item in six out of eight fields that person would have a DI of (3*6)/8 = 2.25. We defined the outcome categories as follows: no disability (DI < 0.50), mild disability (DI 0.50 to 1.00) and severe disability (DI > 1.00) [16]. We included death as a separate outcome category.

### Predictors

Based on previous analyses and the literature we selected candidate predictors [11]. These candidate predictors, as shown in table 1, comprised age and gender beside other demographical, socio-economical, anthropometrical and biochemical variables.

**Table 1:** Baseline characteristics by outcome status after six years.

	No disability (n = 2447)	Mild Disability (n = 878)	Severe disability (n = 781)	Death (n = 921)
Age	63.9 (5.9)	67.5 (6.8)	72.1 (7.7)	75.5 (8.7)

Women	50.8	62.9	77.5	48.9

Partner	74.6	69.6	54.7	53.5

Educational level				

Low	13.1	18.1	31.3	33.6

Intermediate	74.8	72.9	64.3	60.3

High	12.1	9.0	4.4	6.1

Income level				

Low	16.1	20.6	32.2	31.8

Intermediate	49.2	53.4	54.1	52.2

High	34.7	26.0	13.8	16.0

Insurance (public)	46.0	51.5	61.7	61.1

Smoking				

Never	28.9	35.9	44.9	32.0

Formerly	48.0	42.6	36.1	40.8

Currently	23.1	21.5	19.0	27.2

Disability Index	0.11 (0.2)	0.28 (0.1)	0.71 (0.6)	0.71 (0.8)

MMSE	28.2 (1.4)	27.9 (1.5)	27.5 (1.8)	27.0 (2.0)

Self-rated health				

Better	59.0	49.7	40.1	48.1

Same	36.9	41.3	43.3	37.2

Worse	4.0	9.0	16.6	14.7

BMI	25.9 (3.3)	26.7 (3.7)	27.5 (4.0)	26.0 (3.9)

Hypertension	24.9	35.5	39.7	46.4

Depression	29.3	35.5	43.3	34.9

Parkinson's disease	0.0	0.1	1.8	1.1

Diabetes mellitus	3.0	5.1	9.3	11.1

Myocardial infarction	5.6	8.5	8.6	15.9

Stroke	1.2	2.1	5.5	7.8

Respiratory disease	3.1	5.5	6.0	8.9

Osteoarthritis	18.2	30.2	37.0	23.3

Joint complaints	42.6	58.8	71.7	49.0

Morning stiffness	24.8	36.7	51.3	33.7

Falls	9.7	14.7	23.0	22.2

Hearing impairment	2.1	2.9	6.0	11.4

Vision impairment	0.5	1.4	4.3	5.3

Dizziness	6.0	10.1	20.4	14.4

Comorbidities (>1)	11.6	21.9	30.9	28.1

Medication (>2)	16.8	30.4	49.4	48.6

Total cholesterol (mmol/l)	6.7 (1.2)	6.7 (1.1)	6.7 (1.2)	6.4 (1.3)

HDL (mmol/l)	1.4 (0.4)	1.3 (0.3)	1.4 (0.4)	1.3 (0.4)

Data are means with standard deviations for continuous variables and percentages for categorical variables.

### Analysis

After univariable analyses of the individual predictors and disability the significant predictors (p value <0.05) were entered in multivariable analyses. We used polytomous logistic regression analysis with disability status at follow-up as the dependent variable comprising four categories. With the 'no disability' category being the reference category, regression coefficients were estimated for the other categories of mild disability, severe disability and death.

Based on the predictor variables with the highest χ^2 ^in multivariable analyses we finally fitted two multinomial logistic models: one basic model with the three strongest predictors (χ^2 ^> 100): age, gender and baseline disability, and an extended model including the predictors, which have proven to be significantly associated with disability in the literature [11]: joint complaints, self-rated health, cognitive functioning, BMI and hypertension. Interaction terms were included in the models.

The ability of the models to discriminate between different outcomes was studied by estimating the area under the receiver operating characteristics curve (AUC). Based on the regression coefficients of the prediction model we developed a score chart with which outcome probabilities can easily be calculated. The regression coefficients were multiplied by five and rounded to the nearest integer. Probabilities of outcome were calculated with the formulas presented in the appendix.

## Results

### Study population

Subjects who had complete data for sociodemographic factors, life style variables, health conditions and disability status at baseline and complete data for disability at follow-up were included in the analysis (n = 5,027). Of the subjects included, 2,449 (49%) had no disability, 878 (18%) had mild disability, 781 (16%) had severe disability and 919 (18%) had deceased at follow-up after six years. Baseline characteristics of the study sample are presented to outcome status in table 1. In this cohort study less than 5% of the participants were of non-Caucasian origin. Data concerning transitions to other disability status are presented in table 2.

**Table 2:** Transition data

		Functional status at follow up			
Functional status at baseline	Death	Severe disability	Mild disability	No disability	Total
Severe disability	273 (48.1%)	229 (40.3%)	46 (8.1%)	20 (3.5%)	568 (100%)
Mild disability	171 (23.8%)	234 (32.5%	163 (22.6%)	152 (21.1%)	720 (100%)
No disability	475 (12.7%)	318 (8.5%	669 (17.9%)	2277 (60.9%)	3739 (100%)
Total	919 (18.3%)	781 (15.5%	878 (17.5%)	2449 (48.7%)	5027 (100%)

### Predictors

Of the 19 candidate variables that were univariately significantly associated with the outcome eight remained significant (p value < 0.05) at multivariable analysis: age, gender, DI at baseline, cognitive functioning, joint complaints, hypertension, BMI and self-rated health. There was interaction between the variables 'disability index' and 'joint complaints'. Therefore we included this relation as an interaction term in our model as a product of both variables. Odds ratios (OR) with 95% confidence intervals are presented for both the basic and extended model in table 3. An OR on a continuous variable, like the Disability Index, should be interpreted that the OR is related to each one-unit increase on the Disability Index.

**Table 3:** Predictors of disability outcome; basic and extended model

Independent variable	Mild disability		Severe disability		Death	
	**Basic model**	**Extended model**	**Basic model**	**Extended model**	**Basic model**	**Extended model**
**Age (per 10 years)**	2.2 (2.0-2.5)**	2.2 (2.0-2.5)**	3.9 (3.4-4.5)**	4.2 (3.6-4.9)**	7.2 (6.3-8.4)**	7.0 (6.0-8.1)**
**Gender (female)**	1.5 (1.3-1.8)**	1.4 (1.2-1.6)**	2.3 (1.8-2.8)**	2.1 (1.7-2.6)**	0.6 (0.5-0.7)**	0.6 (0.5-0.7)**
**Disability Index**	8.7- (6.4-11.8)**	11.6 (6.3-21.3**)	36.3 (26.6-49.5)**	37.0 (19.9-68.6)**	34.7 (25.4-47.5)**	42.7 (23.5-77.7)**
**MMSE**		0.9 (0.9-1.0)**		0.9 (0.9-1.0)**		0.8 (0.8-0.9)**
**Self-rated health^+^**						
**Same**		1.0		1.0		1.0
**Better**		0.9 (0.7-1.0)		0.7 (0.6-0.9)**		0.8 (0.6-1.0)*
**Worse**		1.7 (1.2-2.3)		2.2 (1.6-3.2)**		2.7 (1.9-4.0)**
**Body mass index**						
**Lower than 25**		1.0		1.0		1.0
**25 to 30**		1.1 (1.0-1.4)		1.3 (1.0-1.6)*		0.8 (0.7-1.0)
**30 or higher**		1.5 (1.2-2.0)		1.9 (1.4-2.6)**		0.9 (0.7-1.2)
**Hypertension**		1.4 (1.1-1.6)**		1.3 (1.0-1.6)*		1.9 (1.5-2.3)**
**Joint complaints**		1.7 (1.4-2.1)**		1.9 (1.4-2.5)**		0.9 (0.7-1.2)
**AUC (95% CI)**	0.67 (0.66-0.69)	0.69 (0.67-0.71)	0.81 (0.79-0.82)	0.82 (0.80-0.83)	0.81 (0.79-0.82)	0.83 (0.81-0.84)

Numbers are odds ratios (95% confidence intervals); * p < .05, ** p < 0.01

The 'no disability' category is used as the reference category

The AUCs of the basic model for the outcomes no disability; mild disability; severe disability and death were 0.83, 0.67, 0.81 and 0.81 respectively. The AUCs for the extended model were slightly higher: 0.85, 0.69, 0.82 and 0.83 respectively.

Score charts for the basic and extended models are presented in tables 4a and 4b respectively. Age and prior disability are the strongest contributors to both models, as reflected by the maximum scores on the MMSE, implying good cognitive functioning, yields a lower probability of disability and death. The contribution of other variables in the extended model was small compared to age and prior disability status.

**Table 4:** a. Score chart basic model and b. Score chart extended model.

a. Score chart basic model			
Predictors	Mild disability	Severe disability	Death
**Age**			
**60**	24	42	60
**70**	28	49	70
**80**	32	56	80
**90**	36	63	90
**Gender**			
**Male**	0	0	0
**Female**	2	4	-3
**Disability Index**			
**0.0**	0	0	0
**0.5**	5	9	9
**1.0**	10	18	18
**1.5**	15	27	27
**2.0**	20	36	36
**2.5**	25	45	45
**3.0**	30	54	54
**Constant**	-34	-59	-76
			
	Sumscore*	Sumscore*	Sumscore*
**b. Score chart extended model**.			
**Predictors**	Mild disability	Severe disability	Death
**Age**			
**60**	24	42	60
**70**	28	49	70
**80**	32	56	80
**90**	36	63	90
**Gender**			
**Male**	0	0	0
**Female**	2	4	-3
**Disability Index**			
**0.0**	0	0	0
**0.5**	5	9	9
**1.0**	10	18	18
**1.5**	15	27	27
**2.0**	20	36	36
**2.5**	25	45	45
**3.0**	30	54	54
**MMSE**			
**20**	0	0	0
**22**	-1	-1	-2
**24**	-2	-2	-4
**26**	-3	-3	-6
**28**	-4	-4	-8
**30**	-5	-5	-10
**Self-rated health**			
**Same**	0	0	0
**Better**	-1	-2	-1
**Worse**	3	4	5
**BMI**			
**< 25**	0	0	0
**25-30**	1	1	1
**≥30**	2	2	3
**Hypertension**	2	2	3
**Joint complaints**	3	3	0
**Constant**	-26	-49	-45
			
	Sumscore*	Sumscore*	Sumscore*

*The probability of each outcome (prognostic score) for an individual can be estimated by adding the scores for each applicable characteristic resulting in a sumscore. The sumscores represents the predicted probability of the outcome category, which may be calculated through the formulas in the appendix

**Figure 1:**
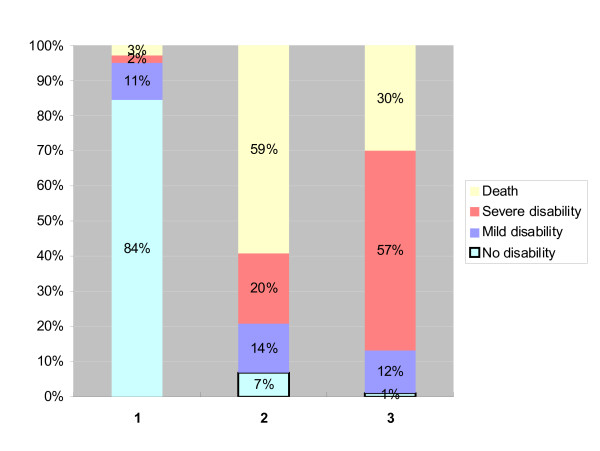
**Probabilities of outcome at six years for three older people with different risk profiles**. Risk profile: 1) male, age 60, DI = 0; 2) male, age 75, DI = 0,75; 3) female, age 70, DI = 1,5.

In Figure 1 the probabilities of different outcomes based on the basic prediction model are given for different baseline profiles: profile one is of a man who is 60 years old and has no baseline disability; profile 2 is of a man who is 75 years old and has mild disability; profile 3 is of a woman who is 70 years and has severe disability at baseline.

## Discussion

This study shows that prior disability and age are the strongest predictors for future disability in older people. Female gender, cognitive functioning, self-rated health as worse than peers, obesity, hypertension and joint complaints contribute to the increase of disability but yield relatively low scores on the chart. In the oldest age groups with prior disability for example, their relevance in predicting future disability status would be negligible. The finding that one can predict future disability with a relatively small set of variables is rather unique. In a recent study a compact frailty index with three components performed as well as a more complex frailty index with five components in predicting falls, disability and mortality [17].

### Strengths and weaknesses

Evaluating single risk factors and targeted single factor interventions do not seem relevant considering the enormous amount of factors already found and their rather small independent association with disability. Therefore we developed a prediction model, which are able to ruling people in or out for preventive interventions. We wanted the model to predict future disability status in a *general *population of older people which comprises people with different functional status, those with and those without prior disability. Furthermore prior disability has shown to be a determinant of subsequent transitions with respect to functional status [18]. We therefore wanted to study how significant this determinant would prove in our prediction model. Although prediction models have been developed for functional decline or disability in older people who were hospitalised because of specific medical conditions, to our knowledge, this is not the case for community-dwelling older people [19]. Therefore, in this study we developed a model and a score chart to predict disability for community-dwelling older people. This method has been used in other areas of research [20,21].

The large cohort from which the prediction rule was derived and the ease with which health care providers may obtain the predictive factors contribute to the strength of this study. The easily obtainability of variables may contribute to a higher implementability of preventive assessment of older people. Another merit of this study is the large ROC areas implicating a good ability of the models to discriminate between different outcomes.

A limitation of this study might be the validity of some of the independent variables. Within six years people might have changed smoking habits, might have developed some chronic diseases or rate their health differently. Still we developed a score chart that estimates the risk of disability at a certain point in time. Another limitation is the lack of validation of the prediction rule in other cohorts although the size of the derivation cohort may compensate for this. In the year 2000 however the first extension cohort of the Rotterdam Study has started comprising over 3000 older people. As longitudinal outcomes are gathered at the moment, external validation can be done in this cohort in the near future. Lastly, we suffered from 37% exclusion of participants because of missing data. These missing data concerns data on possible predictors at baseline or disability data at baseline or follow-up. This exclusion may be selective, but we have no indications that it is.

Prevalence estimates and predictors for disability probably differ depending on the definition of disability used. The definitions that were most often used are: ADL disability, Instrumental ADL (IADL) disability and mobility disability. ADL comprises basic activities like bathing, dressing, toileting, transfer and feeding while IADL also includes activities like transportation, shopping, doing housework and preparing meals. Impaired walking ability, lower extremity disability and homeboundness were all considered as mobility disability [11]. The World Health Organisation defined the term disability in the most recent International Classification of Functioning, Disability and Health more broadly covering impairments (problem in body function or structure), activity limitations (difficulty encountered by an individual in executing a task or action), and participation restrictions (a problem experienced by an individual in involvement in life situations).

As prior disability is the most important predictor and as disability status may be changed for the better by interventive strategies this would imply that there is an opportunity for preventing and treating future disability. A previous study showed that factors related to incident disability (age, gender, self rated health, BMI, joint complaints, depression and medication) are comparable to the factors found in the current extended model on prognosis [22]. This means that interventive strategies can be implemented for preventing as well as treating disability. Although the general idea about disability is that it is irreversible, there are studies that have shown recovery from disability [23,24]. There are several studies on possible strategies including medication or vitamin supplementation, training, home visits and geriatric assessment [2-10]. The care for older people with disability yields high costs. Choosing optimal preventive and therapeutic intervention remains a challenge. To guide these choices more studies on cost-effectiveness are needed as, at present, they are sparse and their results not conclusive [25].

## Conclusion

In this study we were able to predict disability with only a few easily obtainable variables: gender, age and prior disability level. Targeting care and interventive strategies on these predictors would yield the greatest benefits.

## APPENDIX

For the three outcome categories the submodels that constitute the multinomial model can be formulated as follows:

Based on these coefficients found, probabilities for the outcomes can be calculated:

## Competing interests

The authors declare that they have no competing interests.

## Authors' contributions

APV, EWS designed the study; UT and EWS performed the analysis; AH was responsible for data collection; UT was responsible for writing the manuscript; SMABZ, APV, EWS and BWK critically read and approved the manuscript; AH and BWK guaranteed the study. All authors had full access to all the data in the study and final responsibility the integrity of the data, the accuracy of the data analysis and for the decision to submit for publication. All authors read and approved the final manuscript.

## Pre-publication history

The pre-publication history for this paper can be accessed here:

http://www.biomedcentral.com/1471-2318/11/22/prepub
